# Exchanges

**Published:** 1898-08

**Authors:** 


### *BOOK REVIEWS, PAMPHLETS, EXCHANGES.*

---

THE *Living Age* is still a welcome visitor on our exchange table. It always contains much that is worth reading, and each number is complete in itself.

---

MESSRS. SHARP & DOHME have just issued a nice looking and complete price-list. No doubt they will be glad to mail a copy to any physician who requests it.

---

The following pamphlets and reprints have been received:

New Forceps for Intestinal Anastomosis. By Ernest Laplace, M.D., LL.D.

Auto-Intoxication in its Relations to the Diseases of the Nervous System. By Daniel R. Brower, M.A., M.D., Chicago.

Some Observations on the Treatment of Tabes Dorsalis. By Daniel R. Brower, M.A., M.D., Chicago.

Michigan Monthly Bulletin of Vital Statistics.

---

### *EXCHANGES.*

---

THE *Memphis Lancet* is a new monthly journal which is welcomed among our exchanges. To judge whatever of its career by its initial number, success certainly seems assured. The *Lancet* certainly has an able corps of editors, and none the least among them is our good friend Dr. E. C. Ellett. We wish the new journal success.

---

"*Medical Libraries*" is a new publication which has just made its initial appearance. Its chief aim as stated is, "to encourage the founding of medical libraries and medical departments in public libraries wherever the medical profession is fairly organized." This is certainly a worthy project, and should be encouraged in every way possible. We are glad to welcome this publication in our list of exchanges. It is edited by Dr. C. D. Spivak, in Denver, Col.

